# Supplementary figures and images for: Highly Dynamic Gene Family Evolution Suggests Changing Roles for PON Genes Within Metazoa
Source: Genome Biol Evol. 2023 Jan 31;15(2):evad011. doi: 10.1093/gbe/evad011 (PMC9937041; doi:10.1093/gbe/evad011)

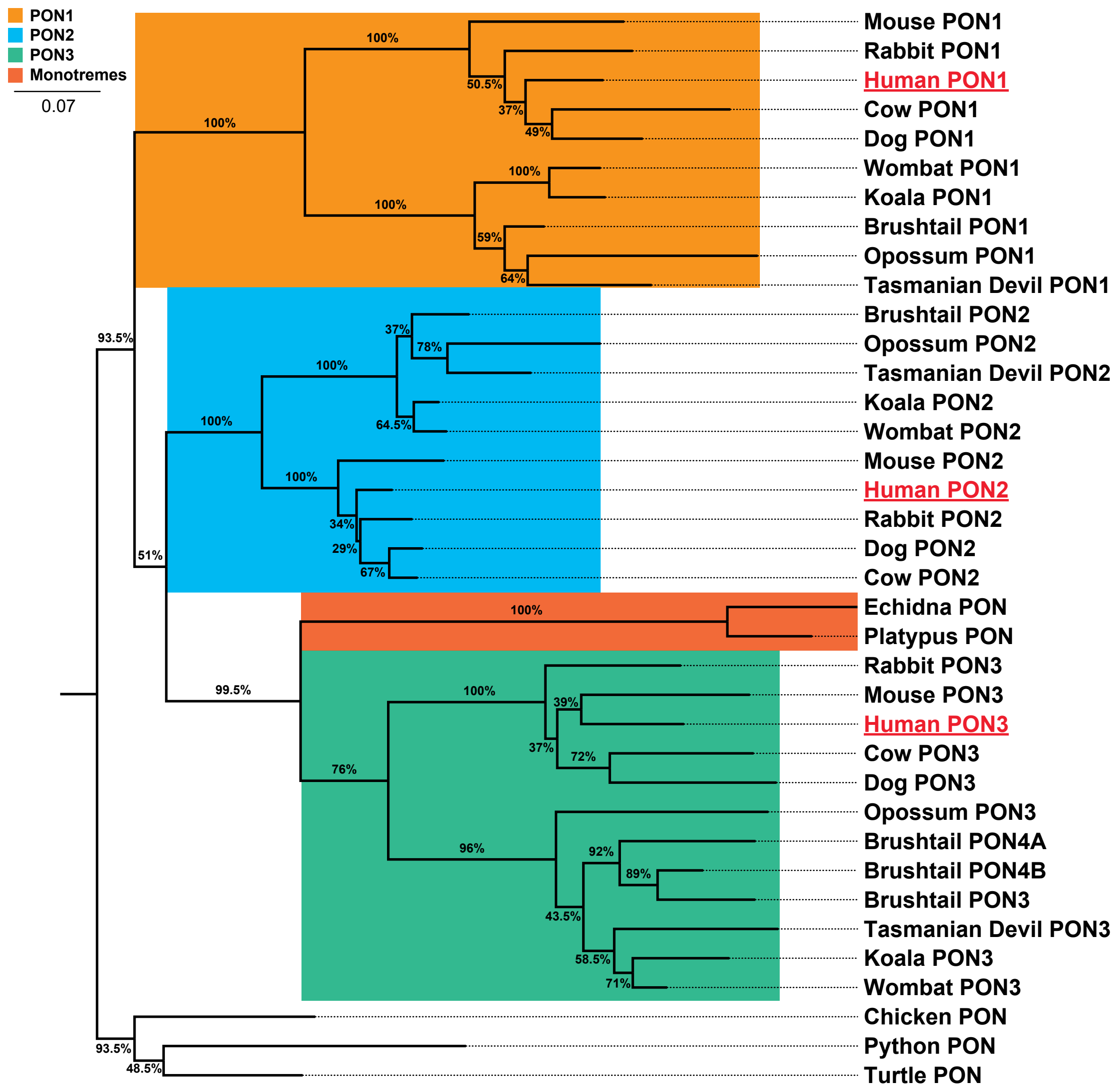

Supplement: evad011_Supplementary_Data [file evad011_supplementary_data.zip › Supplementary Figure 2.pdf]

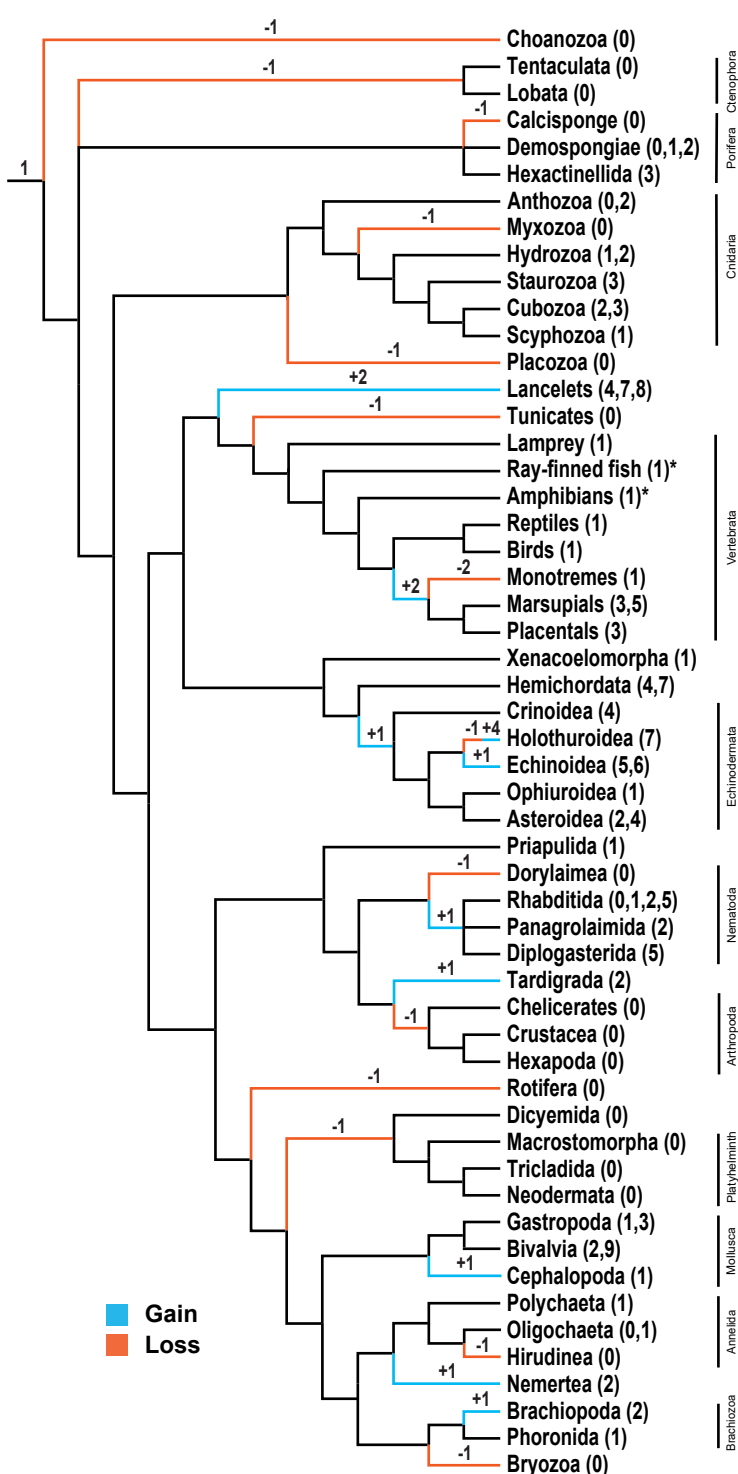

Supplement: evad011_Supplementary_Data [file evad011_supplementary_data.zip › Supplementary Figure 3.pdf]

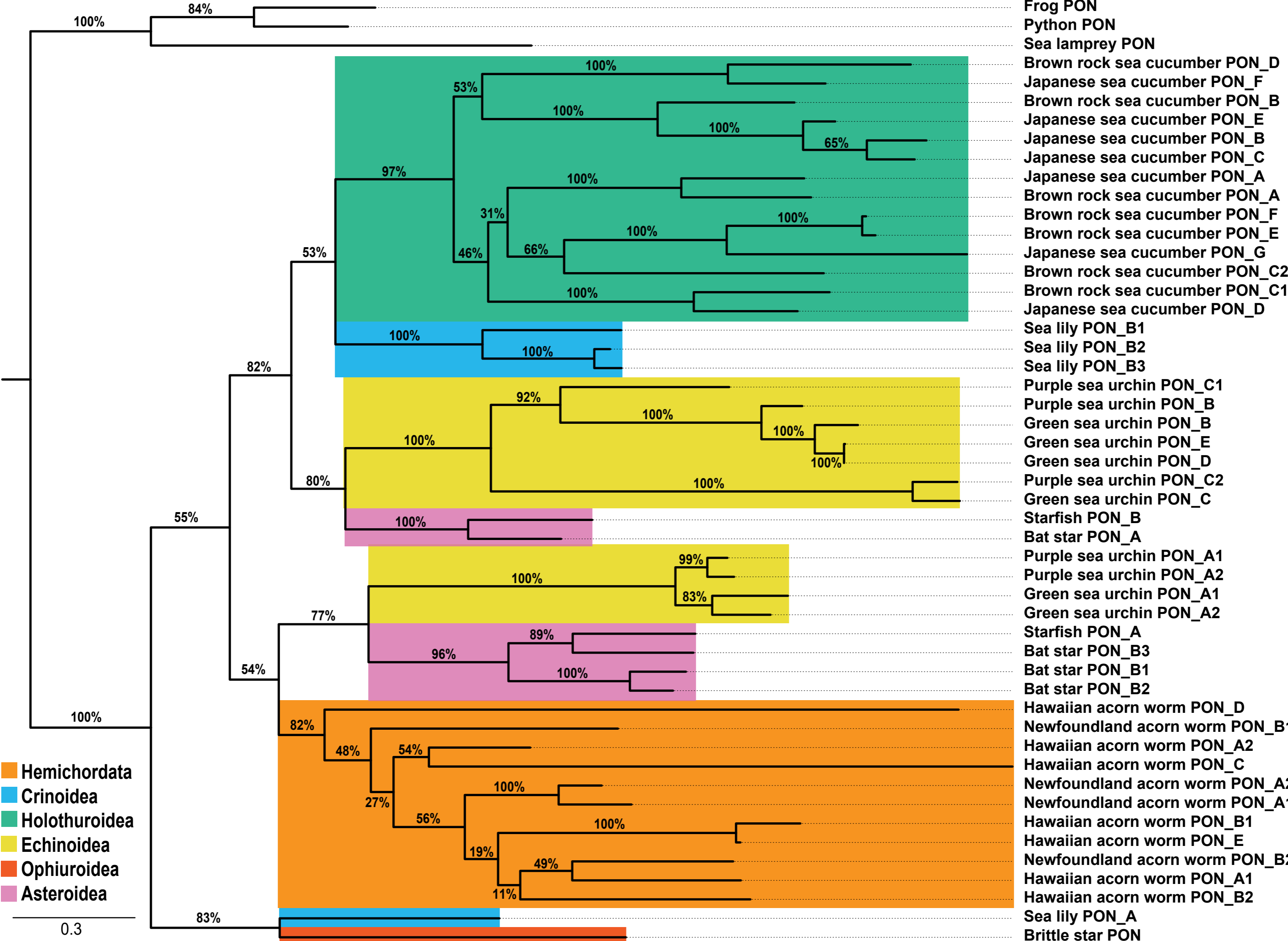

Supplement: evad011_Supplementary_Data [file evad011_supplementary_data.zip › Supplementary Figure 4.pdf]
