# Supplementary material for: Highly Dynamic Gene Family Evolution Suggests Changing Roles for PON Genes Within Metazoa
Source: Genome Biol Evol. 2023 Jan 31;15(2):evad011. doi: 10.1093/gbe/evad011 (PMC9937041; doi:10.1093/gbe/evad011)
Supplement: evad011_Supplementary_Data [file Supplementary Figure 1.pdf › Supplementary Figure 1.pdf]

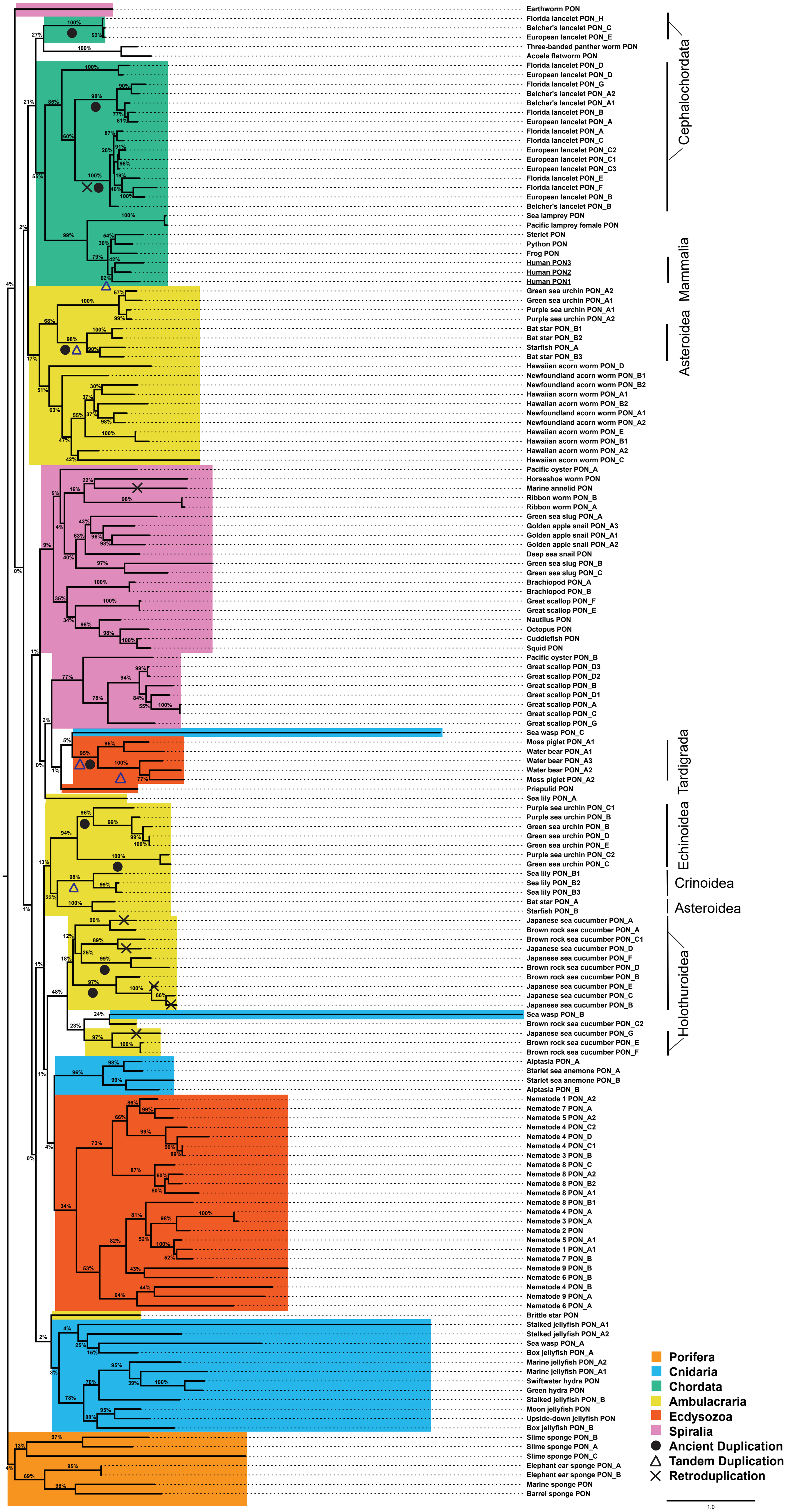

Earthworm PON
Florida lancelet PON_H
Belcher's lancelet PON_C
European lancelet PON_E
Three-banded panther worm PON
Acoela flatworm PON
Florida lancelet PON_D
European lancelet PON_D
Florida lancelet PON_G
Belcher's lancelet PON_A2
Belcher's lancelet PON_A1
Florida lancelet PON_B
European lancelet PON_A
Florida lancelet PON_A
Florida lancelet PON_C
European lancelet PON_C2
European lancelet PON_C1
European lancelet PON_C3
Florida lancelet PON_E
Florida lancelet PON_F
European lancelet PON_B
Belcher's lancelet PON_B
Sea lamprey PON
Pacific lamprey female PON
Sterlet PON
Python PON
Frog PON
Human PON3
Human PON2
Human PON1
Green sea urchin PON_A2
Green sea urchin PON_A1
Purple sea urchin PON_A1
Purple sea urchin PON_A2
Bat star PON_B1
Bat star PON_B2
Starfish PON_A
Bat star PON_B3
Hawaiian acorn worm PON_D
Newfoundland acorn worm PON_B1
Newfoundland acorn worm PON_B2
Hawaiian acorn worm PON_A1
Hawaiian acorn worm PON_B2
Newfoundland acorn worm PON_A1
Newfoundland acorn worm PON_A2
Hawaiian acorn worm PON_E
Hawaiian acorn worm PON_B1
Hawaiian acorn worm PON_A2
Hawaiian acorn worm PON_C
Pacific oyster PON_A
Horseshoe worm PON
Marine annelid PON
Ribbon worm PON_B
Ribbon worm PON_A
Green sea slug PON_A
Golden apple snail PON_A3
Golden apple snail PON_A1
Golden apple snail PON_A2
Deep sea snail PON
Green sea slug PON_B
Green sea slug PON_C
Brachiopod PON_A
Brachiopod PON_B
Great scallop PON_F
Great scallop PON_E
Nautilus PON
Octopus PON
Cuddlefish PON
Squid PON
Pacific oyster PON_B
Great scallop PON_D3
Great scallop PON_D2
Great scallop PON_B
Great scallop PON_D1
Great scallop PON_A
Great scallop PON_C
Great scallop PON_G
Sea wasp PON_C
Moss piglet PON_A1
Water bear PON_A1
Water bear PON_A3
Water bear PON_A2
Moss piglet PON_A2
Priapulid PON
Sea lily PON_A
Purple sea urchin PON_C1
Purple sea urchin PON_B
Green sea urchin PON_B
Green sea urchin PON_D
Green sea urchin PON_E
Purple sea urchin PON_C2
Green sea urchin PON_C
Sea lily PON_B1
Sea lily PON_B2
Sea lily PON_B3
Bat star PON_A
Starfish PON_B
Japanese sea cucumber PON_A
Brown rock sea cucumber PON_A
Brown rock sea cucumber PON_C1
Japanese sea cucumber PON_D
Japanese sea cucumber PON_F
Brown rock sea cucumber PON_D
Brown rock sea cucumber PON_B
Japanese sea cucumber PON_E
Japanese sea cucumber PON_C
Japanese sea cucumber PON_B
Sea wasp PON_B
Brown rock sea cucumber PON_C2
Japanese sea cucumber PON_G
Brown rock sea cucumber PON_E
Brown rock sea cucumber PON_F
Aiptasia PON_A
Starlet sea anemone PON_A
Starlet sea anemone PON_B
Aiptasia PON_B
Nematode 1 PON_A2
Nematode 7 PON_A
Nematode 5 PON_A2
Nematode 4 PON_C2
Nematode 4 PON_D
Nematode 4 PON_C1
Nematode 3 PON_B
Nematode 8 PON_C
Nematode 8 PON_A2
Nematode 8 PON_B2
Nematode 8 PON_A1
Nematode 8 PON_B1
Nematode 4 PON_A
Nematode 3 PON_A
Nematode 2 PON
Nematode 5 PON_A1
Nematode 1 PON_A1
Nematode 7 PON_B
Nematode 9 PON_B
Nematode 6 PON_B
Nematode 4 PON_B
Nematode 9 PON_A
Nematode 6 PON_A
Brittle star PON
Stalked jellyfish PON_A1
Stalked jellyfish PON_A2
Sea wasp PON_A
Box jellyfish PON_A
Marine jellyfish PON_A2
Marine jellyfish PON_A1
Swiftwater hydra PON
Green hydra PON
Stalked jellyfish PON_B
Moon jellyfish PON
Upside-down jellyfish PON
Box jellyfish PON_B
Slime sponge PON_B
Slime sponge PON_A
Slime sponge PON_C
Elephant ear sponge PON_A
Elephant ear sponge PON_B
Marine sponge PON
Barrel sponge PON
Cephalochordata
Mammalia
Asteroidea
Tardigrada
Echinoidea
Crinoidea
Asteroidea
Holothuroidea
Porifera
Cnidaria
Chordata
Ambulacraria
Ecdysozoa
Spiralia
Ancient Duplication
Tandem Duplication
Retroduplication
1.0
